# Intussusception Caused by Heterotopic Pancreas: A Tunisian Case Series of 5 Pediatric Patients

**DOI:** 10.34172/aim.2022.131

**Published:** 2022-12-01

**Authors:** Nahla Kechiche, Dorsaf Makhlouf, Rachida Laamiri, Arije Zouaoui, Salma Mani, Amine Ksiaa, Lasaad Sahnoun, Mongi Mekki, Mohsen Belguith, Abdellatif Nouri

**Affiliations:** ^1^Pediatric Surgery Department, Fattouma Bourguiba Hospital, Monastir, Tunisia

**Keywords:** Heterotopic Pancreas, Intussusception, Surgery

## Abstract

Heterotopic pancreas (HP) is a rare congenital developmental anomaly of the gastro-intestinal tract, defined as the presence of pancreatic tissue found in ectopic sites. Intussusception caused by isolated HP is extremely rare. Pediatric reports concerning this pathology are case reports. Here, we report cases of secondary intussusception, in which conservative treatment failed and surgery was performed. The aim of this review is to study the epidemiologic and clinical aspects of HP in pediatric patients from our institution. We retrospectively collected patients who were treated in the pediatric surgery department for intussusception caused by HP, from January 1986 to November 2018. We investigated five patients, three boys and two girls, aged 5 months to 2 years. The diagnosis was made incidentally during the operation. HP was found in the jejunum in three cases and in the ileum in two cases. HP was removed. The postoperative course was uneventful. Although rare, HP should be included in the differential diagnosis of gastrointestinal diseases, causing secondary bowel intussusception.

## Introduction

 Heterotopic pancreas ( is a rare congenital developmental anomaly of the gastro-intestinal tract, defined as the presence of pancreatic tissue found in ectopic sites, lacking vascular and anatomic communication with the main organ.^1^ Intussusception caused by isolated pancreatic heterotopia is extremely rare.^2^ The real incidence rate of HP is difficult to identify, as in many cases, it is asymptomatic.^3^ Pediatric reports concerning this pathology are case reports.^3^ Here, we report cases of secondary intussusception, in which conservative treatment failed and surgery was performed.

## Case Reports

 We retrospectively collected patients who were treated in the Pediatric Surgery Department, Fattouma Bourguiba Hospital, Monastir, Tunisia, for intussusception caused by HP, from January 1986 to November 2018. We analyzed preoperative diagnosis, ultrasound dataand intraoperative findings. A total of five pediatric patients (three boys and two girls) were admitted to the pediatric surgical department for intussusception. The patients were aged 5, 7, 11, 18 and 24 months.

 The most common symptoms reported were acute abdominal pain in all cases, vomiting in three cases and blood-stained stools in two cases. The classic pediatric triad of abdominal pain, palpable mass and blood-stained stools was found in only two cases.

**Figure 1:**
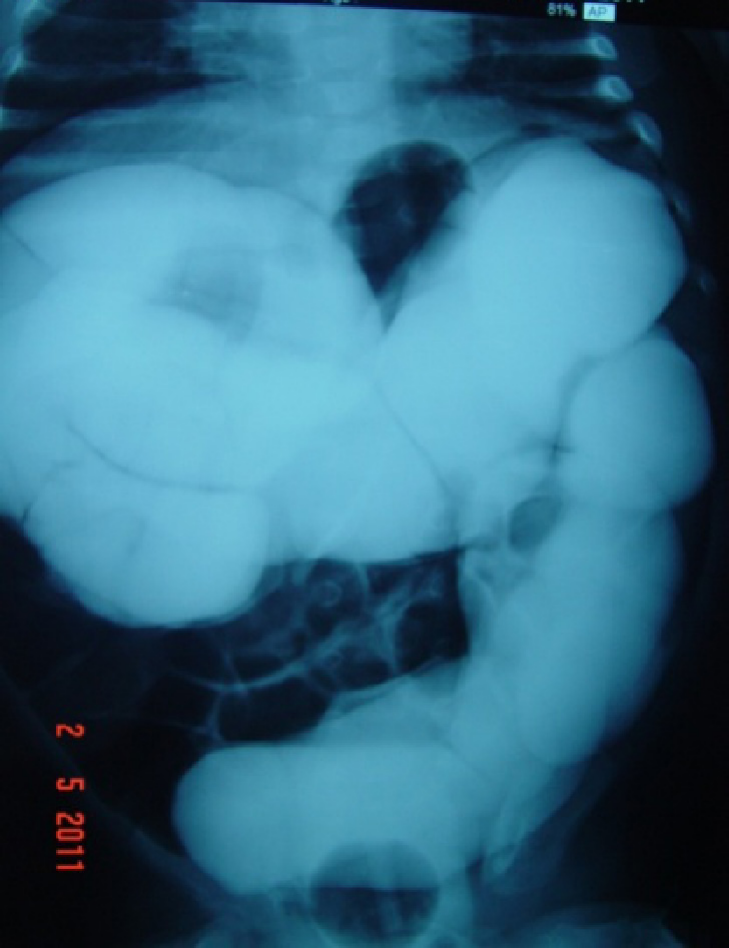
Failure of Hydrostatic Reduction at the Level of the Ileocecal Valve

 Abdominal ultrasound confirmed the diagnosis in all patients. US imaging did not show any suspicious images suggesting the possibility of a secondary intussusception in any of the cases. Hydrostatic enema was our first-line therapy for all patients. Hydrostatic reduction failed at the level of the ileocecal valve in three cases (Figure 1) and at the level of the hepatic flexure in two cases. No specific findings were detected nor suspected while enema reduction was tried.

 Children were operated on after failure of hydrostatic reduction.The indication for surgery was the failure of two attempts of radiological reduction in three cases, and three attempts in two cases.

**Figure 2:**
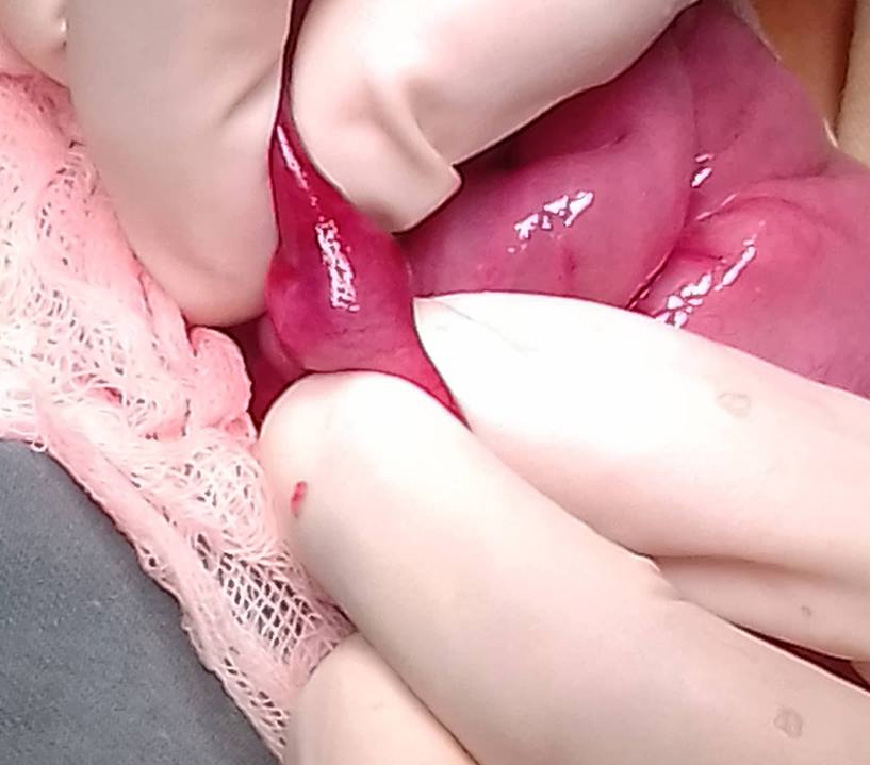
Intraoperative Image of Heterotopic Pancreas in the Jejunum

 Exploratory laparotomywas done and the intussusceptions were manually reduced.The presence of a polypoid formation at the tip of the intussusception was noticed in all cases (Figure 2), the smallest one measuring 10 mm and the largest one 20 mm in diameter, with a mean diameter of 10.5 mm. The HP location was the jejunum in three cases and the ileum in two cases.

**Figure 3:**
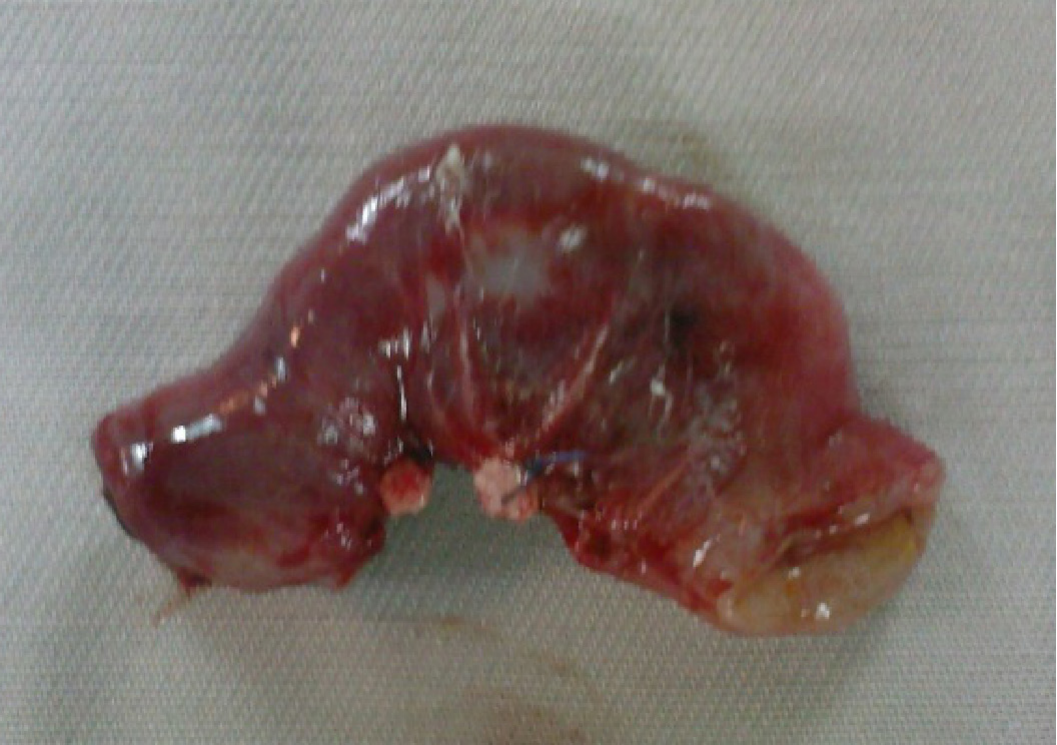
The Surgical Piece

 While exploring the abdomen, we did not find any other intra-abdominal anomaly; in particular, we did not find Meckel’s diverticulum. An intestinal resection was then performed with an end-to-end anastomosis for all patients (Figure 3). The resected segment was sent for histopathological examination. There were no post-operative complications. The histopathological examination confirmed the excised lesion as heterotopic pancreatic tissue. In fact, histological section from the intestine showed acute inflammatory reaction. The nodular mass was made of lobular structures consisting of oval or round cells in acinar and glandular pattern separated by thin fibrous septae. So, in all our patients, the diagnosis of intussusception caused by HP was made.

## Discussion

 HP is represented by an aberrant location of a pancreatic tissue outside the normal pancreas, with no anatomic connection or vascular supply.^4^ The exact pathogenesis remains unknown but several theories have been proposed.^3^ The authors suggest that the most tenable theory implicates is the one stating that during the process of embryonic rotation of pancreatic diverticula (ventral and dorsal pancreatic buds), pancreatic fragments become separated from the main organ and are deposited with no specific order at an ectopic site.^2^ Ectopic pancreas can be seen at any age and particularly between the ages of 30 and 50 years, but pediatric cases are rarely reported.^3-5^ In fact, pediatric reports concerning HP are often simple case reports.^3^ Tanaka et al reported 1 case out of 15 children, Lai reported 6 cases out of 37 patients and Orri et al found 32 cases, with 3 children.^6-8^

 HP is diagnosed incidentally in most cases. It is often asymptomatic.^1^ The patient can become symptomatic when HP is complicated by obstruction, bleeding, malignant transformation or intussusception.^1^

 Orri et al found that 14 of 30 cases were symptomatic and Tanaka et al reported that one third of patients had symptoms.^6,8^ Pediatric reports are generally composed of case reports. HP represents a lead point, telescoping a proximal segment of the digestive tract within the lumen of the adjacent segment.^5^ In large pediatric series, in approximately 2%–12%, a pathological lead point can be the cause of intussusception.^1^ In up to 90% of cases, HP can be found in the upper digestive tract (stomach, duodenum or jejunum).^1^ Authors have reported the sites of HP as follows: duodenum (27.7%), stomach (25.5%), proximal jejunum (15.7%), proximal jejunum (15.9%), Meckel’s diverticulum (5.3%) and ileum (2.8%).^5^ In this study, HP was located in the jejunum in three cases and in the ileum in two.

 Conservative treatment cannot prevent the recurrence of the disease.^3^ In few cases, it can induce pancreatitis or increase the risk for malignant transformation.^3^ So, whenever incidental HP is identified during laparotomy, it should be removed.^3^

 In conclusion, although rare, HP should be included in the differential diagnosis of gastrointestinal diseases, causing secondary bowel intussusception.
